# Structure and Antioxidant Activity of Soy Protein Isolate-Dextran Conjugates Obtained by TiO_**2**_ Photocatalysis

**DOI:** 10.1155/2015/150603

**Published:** 2015-10-01

**Authors:** Bei Jin, Xiaosong Zhou, Bing Li, Caiyan Chen, Xiaosa Zhang, Siqiao Chen

**Affiliations:** ^1^School of Chemistry and Chemical Engineering and Institute of Food Science & Engineering, Lingnan Normal University, Zhanjiang 524048, China; ^2^Engineering Research Center of Starch and Vegetable Protein Processing, South China University of Technology, Ministry of Education, Guangzhou 510640, China; ^3^Development Center for New Materials Engineering and Technology, Lingnan Normal University, Zhanjiang 524048, China

## Abstract

The aim of this study was to investigate the structural characteristics and antioxidant activities of soy protein isolate- (SPI-) dextran conjugates obtained by TiO_2_ photocatalysis treatment. Results revealed that the UV-vis absorption and the fluorescence intensity increased as the photocatalytic power increased (*P* < 0.05). Higher photocatalytic power could promote the extent of glycation and the formation of high molecular weight SPI-dextran conjugates, which were evidenced by free amino group content and sodium dodecyl sulphate-polyacrylamide gel electrophoresis (SDS-PAGE) analysis. The Fourier transform infrared (FT-IR) spectra suggested that the amide I, II, and III bands of SPI were altered by the glycation induced by TiO_2_ photocatalysis. Moreover, significant changes of secondary structure occurred in SPI-dextran conjugates. The *α*-helix, *β*-sheet, *β*-turns, and random coil were changed from approximately 10.6%, 37.9%, 12.9%, and 38.6% to 3.8%, 10.4%, 17.7%, and 68.8%, respectively, after treatment at photocatalytic power of 1000 W. In addition, SPI-dextran conjugates obtained by TiO_2_ photocatalysis treatment exhibited high hydroxyl radical scavenging activity and possessed increased reducing power. All data indicated that TiO_2_ photocatalysis was an efficient method for promoting protein-polysaccharide copolymerisation.

## 1. Introduction

Many modified technologies such as alkylation, esterification, amidination, deamidination, covalent attachment of carbohydrates and fatty acids, thiol-disulfide exchange, and enzymatic modification can effectively improve the functional properties of proteins [1]. Maillard-type protein-polysaccharide conjugates, the product of nonenzymatic browning reaction which is one of the major food protein modifying reactions occurring during thermal food processing, can contribute markedly to the aroma, taste, and colour, as well as to antioxidant potential of stored and processed foods [2]. In addition, Maillard products can be added to foods as functional ingredients to improve the emulsion, gelation, appearance, and texture of food products [3]. However, protein-polysaccharide glycation reaction is time consuming and it is difficult to control the reaction process when using classical glycation reactions, such as dry heating method and wet heating method [1, 4]. Thus, it is necessary to find a novel technology to improve the efficiency of glycation reactions.

Novel physical technologies such as gamma irradiation [5] and pulsed electric field [6] and high intensity ultrasound [7] have been investigated as full or partial alternatives to conventional heat treatment because these technologies can greatly speed up the glycation reaction and improve the functional properties of proteins. There is a growing scientific interest in the influence of photocatalysis on synthesis of compounds with improved properties. Photocatalysis provides a green chemical route for organic functional group transformation under mild conditions. In the past two decades, it has been successfully applied to organic synthesis such as polymerization, hydroxylation of aromatic, oxidation of amine, epoxidation of olefins, and carbonylation [8–12]. In these reactions, no other chemical reagents are introduced and there is no need to control temperature, and the reaction time is shortened. However, the effect of TiO_2_ photocatalysis on the glycation reaction has scarcely been reported. Thus, this study aims to prepare SPI-dextran conjugate induced by TiO_2_ photocatalysis treatment and evaluate the antioxidant activities and the structural changes of the resulting conjugates.

## 2. Materials and Methods

### 2.1. Chemicals

Dextran (MW: 60,000–90,000) was purchased from Chanshou Biological Co., Ltd. (Jiashu Province, China). SPI was obtained from Wonderful Tech. Co. (Shandong Province, China), containing (on dry basis) 6.5% moisture, 1.0% ash, 0.2% lipid, and 90.2% protein (determined by Kjeldahl method, N×6.25). 1, 1-Diphenyl-2-picrylhydrazyl (DPPH), o-phthaldialdehyde (OPA), and 8-anilino-1-naphthalenesulfonic acid (ANS) were purchased from Sigma Chemical Co. (St. Louis, MO, USA). Acrylamide (99%) was purchased from Sigma (Deisenhofen, Germany) and HMF (98%) was from Acros (Geel, Belgium). All other chemicals used were of analytical grade and procured from Merck (Darmstadt, Germany).

### 2.2. Preparation of SPI-Dextran Conjugates

The photocatalytic conjugation was conducted in a 50 mL cylindrical glass vessel fixed in a XPA-II photochemical reactor (Nanjing Xujiang Machine-electronic Plant). The filter system comprises a house-made filter mounted on the lamp to eliminate infrared irradiation and a UV filter which can absorb the light with wavelength less than 400 nm. The mixtures of soy protein and dextran in the weight ratio of 1 : 1 were dissolved in 20 mM potassium phosphate buffer (pH 8). Then, the solution was stirred for 3 h at an ambient temperature until soy protein and dextran completely dissolved. The pH of the solution was adjusted to 8.0 by adding 0.1 N HCl or 0.2 N NaOH. The SPI-dextran solution was ready for the photocatalytic reaction tests. For each test, 20 mL soy protein-dextran solution was placed in a 50 mL jacketed vessel with a constant flow of 4 ± 2°C circulation water at a rate of 0.6 L/min to maintain a sample temperature below 40°C. The SPI-dextran solutions were treated at photocatalytic power of 500 (TP1) and 1000 W (TP2) for 2 h. As a contrast, thermal treated samples were prepared under 40 ± 2°C for 2 h with the same soy protein-dextran mixture (control samples). All samples were kept at 4°C before chemical analysis within 24 h or freeze-dried and then stored at −20°C for further analysis. Further details of photocatalytic reactor could be found in [13].

### 2.3. Spectrophotometric Analyses and Spectrofluorimetry Measurements

The UV-absorbance and browning of SPI-dextran conjugates were measured according to the method of Rao et al. [5] with a slight modification. The absorbance at 294 was measured in 20-fold diluted samples and 420 nm was measured in undiluted samples by a UV-2550 spectrophotometer (Shimadzu, Kyoto, Japan) for detecting UV-absorbance and browning intensity, respectively. Fluorescence measurements were performed using F4500 fluorescence-spectrophotometre (Hitachi Co., Japan). The fluorescence of the glycation products was measured at an excitation wavelength of 290 nm and an emission wavelength 300–450 nm in protein samples of 0.5 *μ*M in 20 mM potassium phosphate buffer (pH 7.2).

### 2.4. Determination of Free Amino Groups Content

The content of free amino groups was determined by the OPA method. The OPA reagent was prepared according to Caillard et al. [14]. OPA (80 mg) was dissolved in 2 mL 95% ethanol and mixed with 50 mL 0.1 M sodium tetraborate buffer solution at pH 9.5, 5 mL of 20% (W/V) SDS, and 0.2 mL 2-mercaptoethanol. The mixtures were then diluted with water to 100 mL for forming the OPA reagent. The OPA reagent was prepared freshly before use. 1 mL OPA reagent was added to 50 *μ*L of the treated soy protein-dextran conjugates and incubated in the dark at 35°C for 2 min, and the absorption at 340 nm was measured immediately in order to obtain the free amino groups.

### 2.5. Determination of Hydroxyl Radical Scavenging Activity and Reducing Power

The hydroxyl radical scavenging activity of the SPI-dextran conjugates was determined according to the method of You et al. [15]. A mix of 600 *μ*L of 1,10-phenanthroline (5.0 mM), 600 *μ*L of FeSO_4_ (5.0 mM), and 600 *μ*L of ethylenediaminetetraacetic acid (EDTA) (15 mM) was mixed with 400 *μ*L of sodium phosphate buffer (0.2 M, pH 7.4). Then 600 *μ*L of samples (2.0 mg/mL) and 800 *μ*L of H_2_O_2_ (0.01%) were added. The mixture was incubated at 37°C for 60 min, and the absorbance was measured at 536 nm (UV754, Xianjian Scientific Instrument Co., Shanghai, China). Results were determined using the following equation:

(1) Hydroxyl  radical  scavenging  activity  %=As−A0×100Ac−A0,

where *A*_*s*_ is the absorbance of the sample, *A*_0_ is the absorbance of the blank solution using distilled water instead of sample, and *A*_*c*_ is the absorbance of a control solution in the absence of H_2_O_2_.

The reducing power of conjugation was determined according to the method of Zheng et al. [16] with some modification. Briefly, 2.0 mL of sample was mixed with 2.0 mL of 0.2 M sodium phosphate buffer (pH = 6.6) and 2.0 mL of 1% (w/v) potassium ferricyanide. The mixture solution was incubated at 50°C for 20 min followed by the addition of 2.0 mL of 10% trichloroacetic acid. The mixtures were centrifuged at 3000 r/min for 10 min. 2.0 mL of the supernatant was collected and mixed with 2.0 mL of distilled water and 0.4 mL of 0.1% (w/v) FeCl_3_. After standing at room temperature for 10 min, the absorbance of the reaction mixture was measured spectrophotometrically at 700 nm. An equivalent volume of distilled water instead of the sample was used as the blank. Increased absorbance of the reactions mixture indicated increased reducing power.

### 2.6. Electrophoresis

SDS-polyacrylamide gel electrophoresis (SDS-PAGE) was examined by vertical gel electrophoresis equipment (Mini-Protean II; Bio-Rad Laboratories, Richmond, CA) [17]. The sample (10 *μ*g) was added with laemmli buffer in the presence of 2% *β*-mercaptoethanol and heated at 100°C for 5 min before loading into gel. The electrophoresis was carried out at a constant current of 15 mA using 4% stacking gel and 10% running gel. After separation, Gels were stained with 0.2% Coomassie Brilliant Blue R-250 in 25% methanol and 10% acetic acid. Destaining was conducted with a solution of 40% methanol and 10% acetic acid. Then, the gels were scanned and analyzed.

### 2.7. Fourier Transform Infrared (FT-IR) Measurement

The infrared analysis was performed using the FT-IR technique according to the method described by Gao et al. [18] with a slight modification. FTIR spectra were measured on a FT-IR spectrometer (NICOLET NEXUS470, DTGS) with a 4 cm^−1^ resolution and 32 scans between wavenumbers of 4000 cm^−1^ and 400 cm^−1^. The freeze-dried samples were prepared as KBr disks with 1 mg of the samples in 100 mg of KBr. Background noise was corrected with pure KBr data. The spectra were averaged and smoothed, and their baselines were calibrated with the Spectra Manager software (Jasco Inc., Easton, MD, USA).

### 2.8. CD Spectra Measurements and CD Spectra Analysis

The secondary structure of proteins was determined by CD at 25°C in the far UV (from 190 to 250 nm) using a CD6 Jobin-Yvon dichrograph. Spectra were recorded at a protein concentration of 0.5 mg/mL in 20 mM PBS (pH 7.2) after the centrifugation at 5,000 ×g to remove any insoluble residue using a 2 mm path length quartz cuvette. Secondary structure was estimated using the CONTIN software. Four secondary structures: *α*-helix, *β*-sheet, *β*-turns, and unordered coil, were calculated. Data were the means of triplicate measurements.

### 2.9. Statistical Analysis

All analyses were done in triplicate, and data are reported as means ± standard deviation. Differences between the variables were tested for significance by one-way ANOVA accompanied with Tukey's post hoc test using Origin 7.5. A value of *P* < 0.05 was considered significant.

## 3. Results and Discussion

### 3.1. Changes in *A*_294_, Browning Intensity and Fluorescence Intensity

**Figure 1:**
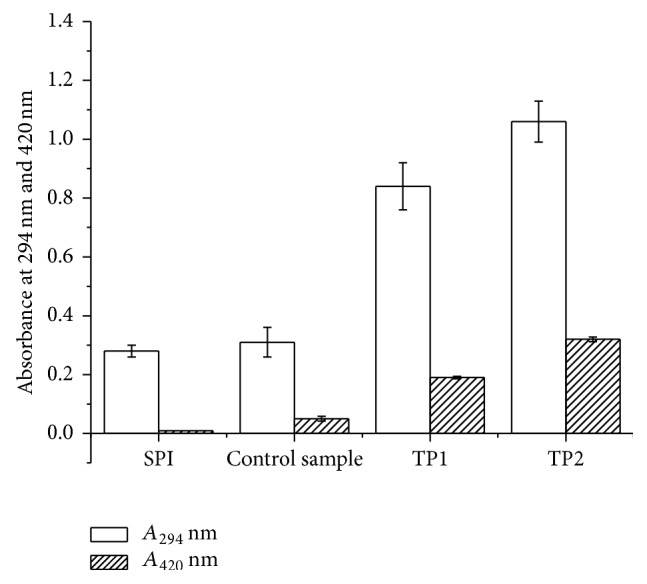
*A*_294_ and browning intensity analysis of SPI and SPI-dextran solution treated by heat and TiO_2_ photocatalysis (control sample, TP1 and TP2). The data with different lowercase letters in the same test are significantly (*P* < 0.05) different.

The changes of *A*_294_ and browning intensity of SPI-dextran model solution with different photocatalytic power levels are shown in Figure 1. Both *A*_294_ and browning changed significantly (*P* < 0.05) at photocatalytic power of 500 and 1000 W within 2 h. The *A*_294_ of SPI-dextran solution increased from approximately 0.28 to 0.84 and 1.06 as photocatalytic power of 500 and 1000 W, respectively; in the case of *A*_420_, it increased from approximately 0 to 0.21 and 0.36 at photocatalytic power of 500 and 1000 W, respectively. The brown pigment development, indicated by *A*_420_, coincided with the colourless intermediate formation evidenced by increased *A*_294_, suggesting that the brown pigments were formed in parallel to the generated intermediate products. However, higher increase in *A*_294_, comparing to increase in *A*_420_, suggests high photocatalytic power could dominate the early stage of the Maillard reaction. In general, we hoped to reduce the formation of melanoidins and increase the yield of uncolored products with better functional properties.

**Figure 2:**
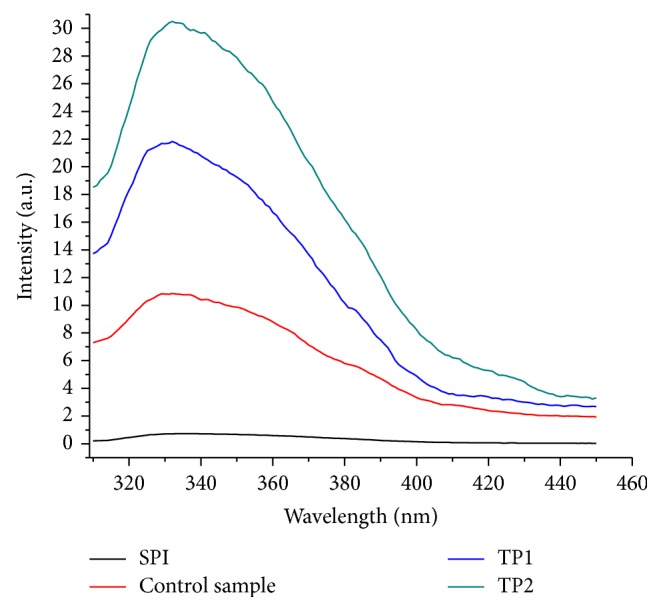
Fluorescence analysis of SPI and SPI-dextran solution treated by heat and TiO_2_ photocatalysis (control sample, TP1 and TP2).

The Maillard reaction is also associated with the development of fluorescent compounds. In the present study, formation of fluorescent compounds was observed at photocatalyzed SPI-dextran solution suggesting the formation of the resulting conjugate. Photocatalyzed samples have shown increased fluorescence with maximum at about 331 nm when excited at 290 nm originating from glycation products. Fluorescence of Maillard products was the highest in SPI-dextran solution at photocatalytic power of 1000 W (Figure 2) and in accordance with spectrophotometric properties of tested samples. The results suggested that photocatalysis can lead to breakage of glycosidic bonds in dextran and so more number of carbonyl groups are available for formation of SPI-dextran conjugate, similar to those induced by irradiation resulting in obvious increase in UV-absorbance and fluorescence [5].

### 3.2. Changes of Free Amino Groups Content

**Figure 3:**
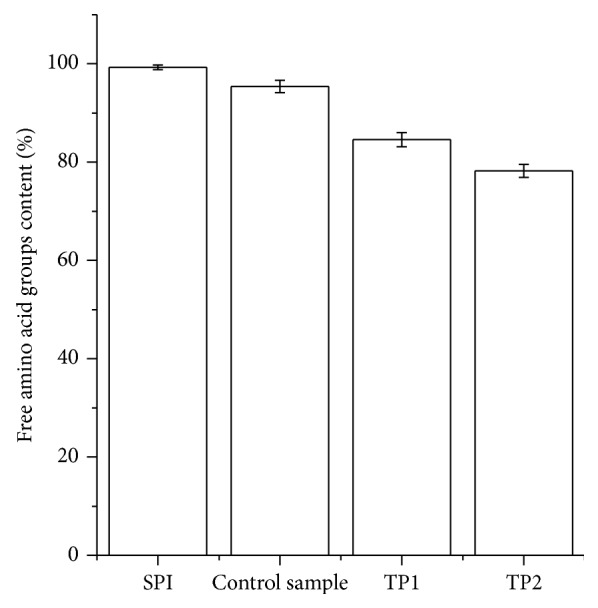
Changes in free amino groups content of SPI and SPI-dextran solution treated by heat and TiO_2_ photocatalysis (control sample, TP1 and TP2). The data with different lowercase letters in the same test are significantly (*P* < 0.05) different.

Changes in free amino group content of SPI-dextran solution after different photocatalytic power treatments are depicted in Figure 3. The free amino groups content in the SPI-dextran model system at photocatalytic power of 500 and 1000 W was reduced by 14.7% and 21.1%, respectively, while little changes were observed in control tests. These results suggested that photocatalysis at higher power could promote the interaction between free amino groups of SPI and carbonyl group of dextran to form glycated product. From the results, it is obvious that the decrease in free amino group was in accordance with only a small increase in browning at 420 nm (Figure 1), which was in accordance with the report of Xu et al. [19] who reported that free amino groups in *β*-conglycinin-dextran model system was decreased during the Maillard reaction. Therefore the effect of photocatalysis on the browning reaction and the loss of free amino groups are actually ideal for the glycation of protein and polysaccharides.

### 3.3. Changes in Hydroxyl Radical Scavenging Activity and Reducing Power

**Figure 4:**
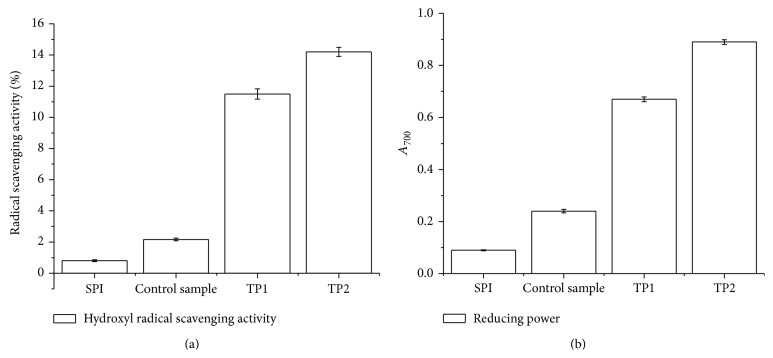
Changes in hydroxyl radical scavenging activity (a) and reducing power (b) of SPI and SPI-dextran solution treated by heat and TiO_2_ photocatalysis (control sample, TP1 and TP2). The data with different lowercase letters in the same test are significantly (*P* < 0.05) different.

The hydroxyl radical scavenging abilities and reducing power were used as the standards to assess the antioxidative activity of SPI-dextran solution and the result is shown in Figure 4. Hydroxyl radical-scavenging activity ratio of SPI-dextran conjugates was significantly increased from approximately 0.81% to 11.5% and 14.2% at photocatalytic power of 500 and 1000 W, respectively. However, no significant changes were found in the control tests within all the reactions (*P* > 0.05). The reducing power of SPI-dextran conjugates (Figure 4(b)) showed similar trends with those of radical scavenging activity. The results indicate that SPI-dextran conjugates were free radical inhibitors and reducing agents as well as their concentration increased with photocatalytic power. Our findings are in agreement with an earlier report on antioxidant activity of other model systems, as a result of conjugates induced by gamma radiation in nisin model system [20].

### 3.4. Changes in Protein Pattern

**Figure 5:**
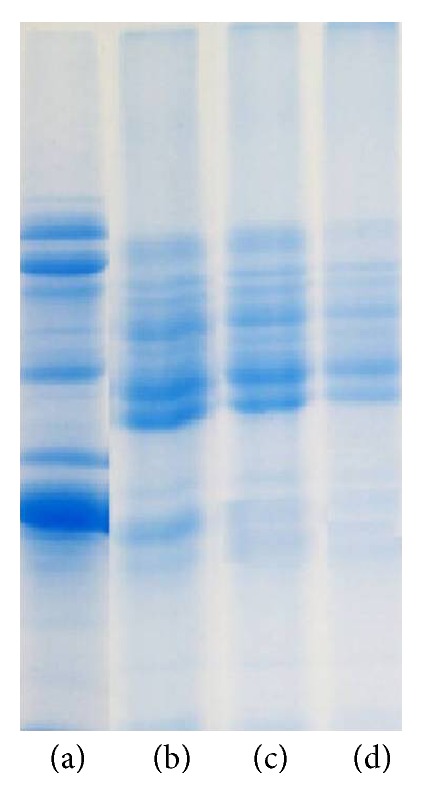
SDS-PAGE of protein patterns of SPI and SPI-dextran conjugates obtained by heat and TiO_2_ photocatalysis. ((a) SPI; (b) control sample; (c) TP1; (d) TP2).

SDS-PAGE was performed to further confirm the covalent coupling of dextran and SPI after different photocatalytic power treatment as shown in Figure 5. Initially, the dextran components in the reaction mixtures were not obtained by Coomassie blue staining; only the soy protein subunits were observed. The soy protein subunit bands in the control test are slightly lighter than the untreated sample. On the other hand, photocatalytic power exhibited a pronounced effect on crosslinking. More soy protein subunits disappeared as the photocatalytic power intensity increased. The new faint bands with much higher molecular weight on the top of the gel were observed in SPI-dextran conjugates by TiO_2_ photocatalysis, indicating that a large amount of new amino groups was exposed and reacted with carbonyl groups to form Maillard-based aggregates gradually because most noncovalent interactions are generally disrupted in SDS-PAGE; this was shown in the browning and free-amino analysis. These materials and the high molecular weight may be related to its reducing power and hydroxyl radical-scavenging activity. Similar SDS-PAGE was found by Zhang et al., who reported that covalent conjugating compounds were produced in *β*-conglycinin-dextran model system treated by heating [21].

### 3.5. FT-IR Analysis

**Figure 6:**
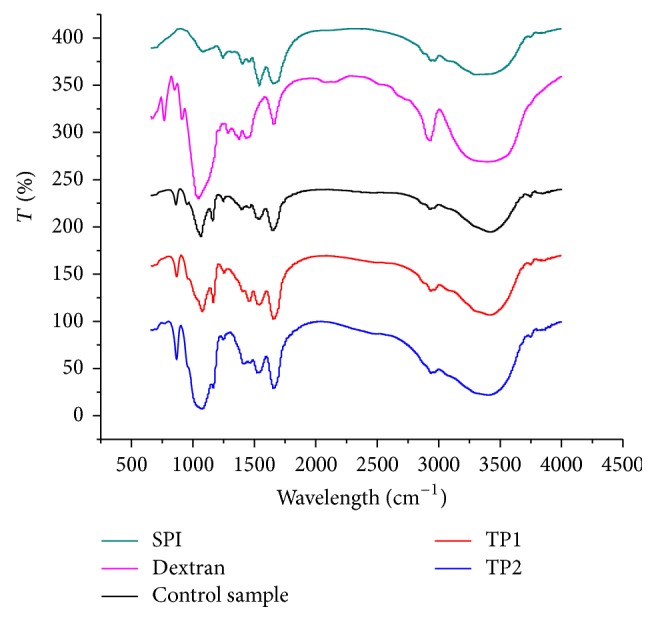
Infrared spectra of SPI, dextran, and SPI-dextran conjugates obtained by heat and TiO_2_ photocatalysis (control sample, TP1 and TP2).

The spectroscopic analysis of polymeric molecules, including proteins, is complex due to the molecular vibrations arising from numerous atoms. FT-IR spectroscopy is a particularly useful technique for the study of protein-carbohydrate systems, as there are several readily identifiable regions of the mid-infrared spectrum where the chemical fingerprints of carbohydrates and proteins do not overlap significantly. As shown in Figure 6, a major band at 3299 cm^−1^ was observed in the spectra of SPI. This peak was denoted to the stretching of hydrogen-bonded O–H groups. Meanwhile, SPI exhibited two characteristic bands at 1649 (amide I, C=O stretching) and 1535 cm^−1^ (amide II, N–H bending) [22]. For SPI-dextran conjugates, the regions of 1649 cm^−1^ and 1535 cm^−1^, which are referred to as C–O and C–N stretching from amide I and II, were modified by the Maillard reaction (Figure 6). Compared with intact SPI, the SPI-dextran conjugates at at photocatalytic power of 1000 W rendered the lowest intensity. It might be expected that the –OH group in dextran and the amino groups in SPI are consumed in reaction mixture under TiO_2_ photocatalysis treatment. Su et al. [22] found a gradual decrease in the intensity of the bands at 1600–1400 cm^−1^ during the Maillard reaction between carboxymethyl cellulose (CMC) and soy protein isolate (SPI). For carbohydrates, a series of overlapping peaks located in the region of 1180–953 cm^−1^ results from vibration modes such as the stretching of C–C and C–O and the bending mode of C–H bonds. These are often referred to as the “saccharide” bands and are the most intense bands in the mid-infrared spectrum. These absorptions are weak in the spectra of most proteins [23]. The absorptions in the region of 1180–953 cm^−1^ were stronger in SPI-dextran conjugate at the different photocatalytic power and the control sample than in SPI and weaker than dextran, indicating that there seemed to be a saccharide attached to the SPI. In addition, in proteins, there is an amide III band at 1300–1200 cm^−1^. This band is known to be very complex and mainly arises from C–N stretching and N–H deformation. The entire spectral features of the amide III band for the SPI-dextran solution at 500 and 1000 W (Figure 6) showed a decrease in intensity compared to SPI and control sample. It might be expected that the chemical changes accompanying the Maillard reaction in SPI would lead to several changes in the IR spectrum as a result of the consumption of some functional groups (–NH_2_).

### 3.6. Secondary Structure Changes

The secondary structures of dispersions of SPI and SPI-dextran conjugates under classical heating or TiO_2_ photocatalysis conditions were measured, and the detailed data of *α*-helix, *β*-sheet, *β*-turns, and random coil levels are shown in Table 1. Native dispersions of SPI contained approximately 10.6% *α*-helix, 39.9% of *β*-sheet, 10.9% of *β*-turns, and 38.6% of random coil. Significant changes were observed after it was treated at different photocatalytic power. The *α*-helix, *β*-sheet, *β*-turns, and random coil were changed to approximately 5.9%, 20.3%, 14.5%, and 59.8% after being treated at 500 W. With increasing photocatalytic power, *α*-helix, *β*-sheet, and random coil changed remarkably to approximately 3.8%, 10.4%, and 68.8% at 1000 W, whereas *β*-turns had only a slight change. However, slight but not significant changes were found in the control tests. Higher photocatalytic power is more effective in changing the secondary structure of soy protein in the presence of dextran. Increasing photocatalytic power can provide a larger degree of glycation, as also evidenced by the loss of free amino groups, so that more dextran was conjugated to soy protein, resulting in an increased content of disordered structure. It proved that unordered structure was dominant in the secondary structure for the glycated SPI. These changes suggested that conjugating polysaccharides to protein by covalent bonds (as shown by SDS-PAGE) can lead to conformational changes to the secondary structure of protein-polysaccharide conjugates.

## 4. Conclusion

SPI-dextran conjugate was successfully formed by TiO_2_ photocatalysis treatment. In particular, higher photocatalytic power prompted glycation. Significant increases were found in the antioxidant properties of SPI by its conjugation with dextran after TiO_2_ photocatalysis treatment. Moreover, the measured increase in antioxidant activity coincided with an increase in the UV-vis absorption and fluorescence intensity and a decrease in free amino group content. The SDS-PAGE showed that a new high molecular mass was produced and original soy protein subunits disappeared after the conjugation induced by TiO_2_ photocatalysis. The spectroscopic analysis using the FT-IR technique indicated that the amide I, II, and III bands of SPI were modified by dextran after TiO_2_ photocatalysis treatment. Additionally, CD spectroscopy result further confirmed that glycation could change soy protein secondary structure at higher TiO_2_ photocatalytic power. These results suggested that TiO_2_ photocatalysis could potentially be applied as an effective way for forming protein and polysaccharide conjugates. Further work on conjugation between SPI and polysaccharides with TiO_2_ photocatalysis treatment will be conducted to elucidate the conjugating mechanism.

## Figures and Tables

**Table 1:** Secondary structure distribution of SPI and SPI-dextran conjugates obtained by heat and TiO_2_ photocatalysis (control sample, TP1 and TP2). Values with the same lowercase letters are not significantly different (*P* > 0.05).

	*α*-helix (%)	*β*-sheet (%)	*β*-turns (%)	Random coil (%)
SPI	10.6 ± 0.2	37.9 ± 0.4	12.9 ± 0.1	38.6 ± 0.3
Control sample	10.3 ± 0.2	31.5 ± 0.3	18.3 ± 0.2	40.1 ± 0.4
TP1	5.9 ± 0.1	20.3 ± 0.2	14.5 ± 0.4	59.8 ± 0.4
TP2	3.8 ± 0.1	10.4 ± 0.2	17.7 ± 0.4	68.8 ± 0.5
